# Viral species differentially influence macronutrient preferences based on honey bee genotype

**DOI:** 10.1242/bio.059039

**Published:** 2022-09-30

**Authors:** Hannah J. Penn, Michael D. Simone-Finstrom, Lilia I. de Guzman, Philip G. Tokarz, Rachel Dickens

**Affiliations:** ^1^ USDA ARS Sugarcane Research Unit, 5883 Usda Rd., Houma, LA, USA 70360-5578; ^2^ USDA ARS Honey Bee Breeding, Genetics and Physiology Laboratory, 1157 Ben Hur Rd., Baton Rouge, LA, USA 70820-5502

**Keywords:** Chronic bee paralysis virus, Deformed wing virus, Honey bee, Host-pathogen interactions, Lipid, Protein

## Abstract

Food quantity and macronutrients contribute to honey bee health and colony survival by mediating immune responses. We determined if this held true for bees injected with chronic bee paralysis virus (CBPV) and deformed wing virus (DWV), two common honey bee ssRNA viruses. Pollen-substitute diet and syrup consumption rates and macronutrient preferences of two *Varroa*-resistant stocks (Pol-Line and Russian bees) were compared to *Varroa*-susceptible Italian bees. Bee stocks varied in consumption, where Italian bees consumed more than Pol-Line and Russian bees. However, the protein: lipid (P:L) ratios of diet consumed by the Italian and Russian bees was greater than that of the Pol-Line bees. Treatment had different effects on consumption based on the virus injected. CBPV was positively correlated with syrup consumption, while DWV was not correlated with consumption. P:L ratios of consumed diet were significantly impacted by the interaction of bee stock and treatment, with the trends differing between CBPV and DWV. Variation in macronutrient preferences based on viral species may indicate differences in energetic costs associated with immune responses to infections impacting different systems. Further, virus species interacted with bee genotype, indicating different mechanisms of viral resistance or tolerance among honey bee genotypes.

## Teaser

**Summary:** Honey bee stocks interact differentially with two ssRNA viruses to alter the ratio of protein and lipid in foraged diet and the amount of consumed sugar syrup quantities.

## INTRODUCTION

Malnutrition is a key concern for the health of honey bee (*Apis mellifera* Linnaeus, Hymenoptera: Apidae) colonies both directly via lack of food resources (DeGrandi-Hoffman et al., 2016) and indirectly through disease susceptibility (Alaux et al., 2010; DeGrandi-Hoffman et al., 2010). Honey bees require pollen and nectar as their primary food sources, with pollen being the main source of both protein and lipids (Brodschneider and Crailsheim, 2010). The availability and quality of food resources like pollen can have colony-wide health implications since nurse bees require pollen for hypopharyngeal gland development to feed developing larvae (Keller et al., 2005; Roulston and Cane, 2000). Honey bee colonies with access to supplemental or high-quality pollen exhibit increased hypopharyngeal gland size (Corby-Harris et al., 2018), greater brood production (Ricigliano et al., 2018), and increased survival when faced with disease threats (Erler and Moritz, 2016; Pasquale et al., 2013).

Honey bee colonies harbor various pathogens, parasites, and pests, most of which contribute to colony loss to varying degrees due to their impact on the physiology and behavior of individual honey bees (Alaux et al., 2012; Barroso-Arévalo et al., 2019; Dainat et al., 2012; Faurot-Daniels et al., 2020; Holt et al., 2013; Natsopoulou et al., 2016). The precise impact of the parasites and pathogens also depends on honey bee genetics, as stocks of honey bees respond differently when exposed to *Nosema spp.* (Goblirsch et al., 2013; Malone et al., 1995), tracheal mites (*Acarapis woodi* Rennie) (de Guzman et al., 2002), or *Varroa* mites (*Varroa destructor* Anderson & Trueman) (Tarpy et al., 2007; Wilfert et al., 2016). These genetic differences may also result in differential foraging behaviors (Gary et al., 1978; Guzman-Novoa and Gary, 1993) and parasite and pathogen tolerance (Gary et al., 1978; Guzman-Novoa and Gary, 1993; Ihle et al., 2010; Khongphinitbunjong et al., 2016; Locke et al., 2014; Penn et al., 2022a).

Nutrition may play a key role in mediating honey bees' immune responses to pathogens and parasites via consumption rates or macronutrient preferences (Alaux et al., 2010; Harwood et al., 2019). Pollen has been shown to enable bees to maintain function while under stress from *Nosema spp.* infection (Azzouz-Olden et al., 2018; Jack et al., 2016) or *Varroa* mite infestation (Annoscia et al., 2017). Additionally, honey bee foragers exhibit preferences for plant products such as pollen and nectar (Hawkins et al., 2015), potentially as a way to obtain particular macronutrients (Cook et al., 2003), self-medicate at the individual level (Erler and Moritz, 2016), or to socially-medicate at the colony level (Penn et al., 2022b; Simone-Finstrom and Spivak, 2012; Spivak et al., 2019). For instance, honey bees exposed to fungal pathogens preferentially foraged on lipid-rich pollens, which increased survival of infected individuals (Foley et al., 2012). Access to pollen with antimicrobial profiles or added protein resources has been shown to alter bee immune responses to *Nosema spp.* infections, increasing survival and diminishing spore loads (Gherman et al., 2014; Rinderer and Dell Elliott, 1977). High-quality pollen consumption and related increases in protein availability can also reduce deformed wing virus (DWV) titers (DeGrandi-Hoffman et al., 2010) and decrease mortality induced by other single-stranded RNA (ssRNA) viruses like Israeli acute paralysis virus (IAPV) (Dolezal et al., 2019).

Parasitism or pathogen infection itself may determine the foraging decisions made by the impacted insect (Lee et al., 2006). Caterpillars that experienced bacterial infections reduced overall feeding and carbohydrate intake (Povey et al., 2014) while caterpillars experiencing parasitoidism reduced protein consumption (Mason et al., 2014). In red imported fire ant foragers, infection with an ssRNA virus decreased overall foraging levels and altered the feeding preferences from protein- and lipid-rich to carbohydrate-rich foods (Hsu et al., 2018). Honey bee foragers infected with *Nosema apis* (Zander) were more likely to visit artificial flowers with only nectar whereas uninfected bees foraged at similar rates on both nectar and pollen (Lach et al., 2015). This increased preference for sugar may suggest increased hunger or heightened responsiveness to sugar content by infected foragers (Chen et al., 2014; Kralj and Fuchs, 2010; Martín-Hernández et al., 2011; Mayack and Naug, 2009; Naug and Gibbs, 2009). Such changes in foraging behavior may be related to trade-offs in insect immune response and the ability to synthesize, store, and metabolize lipids or protein, resulting in decreased food consumption or host manipulation by parasites and pathogens (Adamo et al., 2010; Bernardo and Singer, 2017; Li et al., 2018; Shikano and Cory, 2016).

However, we do not know if honey bee consumption of pollen and nectar or macronutrient preferences change in direct response to immune challenges from common ssRNA viruses (Chen et al., 2006a; Dainat et al., 2012; de Miranda and Genersch, 2010; Lanzi et al., 2006). Further, we do not know if virus-induced preferences differ between bee stocks with varying susceptibilities to the virus-vectoring parasitic *Varroa* mite (susceptible: Italian, resistant: Pol-line and Russian) (Danka et al., 2016; de Guzman et al., 2007; Di Prisco et al., 2016; Nazzi et al., 2012). Therefore, the overarching goal of this study was to determine if infection with different viruses alters honey bee diet consumption or macronutrient preferences and if this occurs to a similar extent in susceptible and resistant bee stocks.

**Fig. 1.**
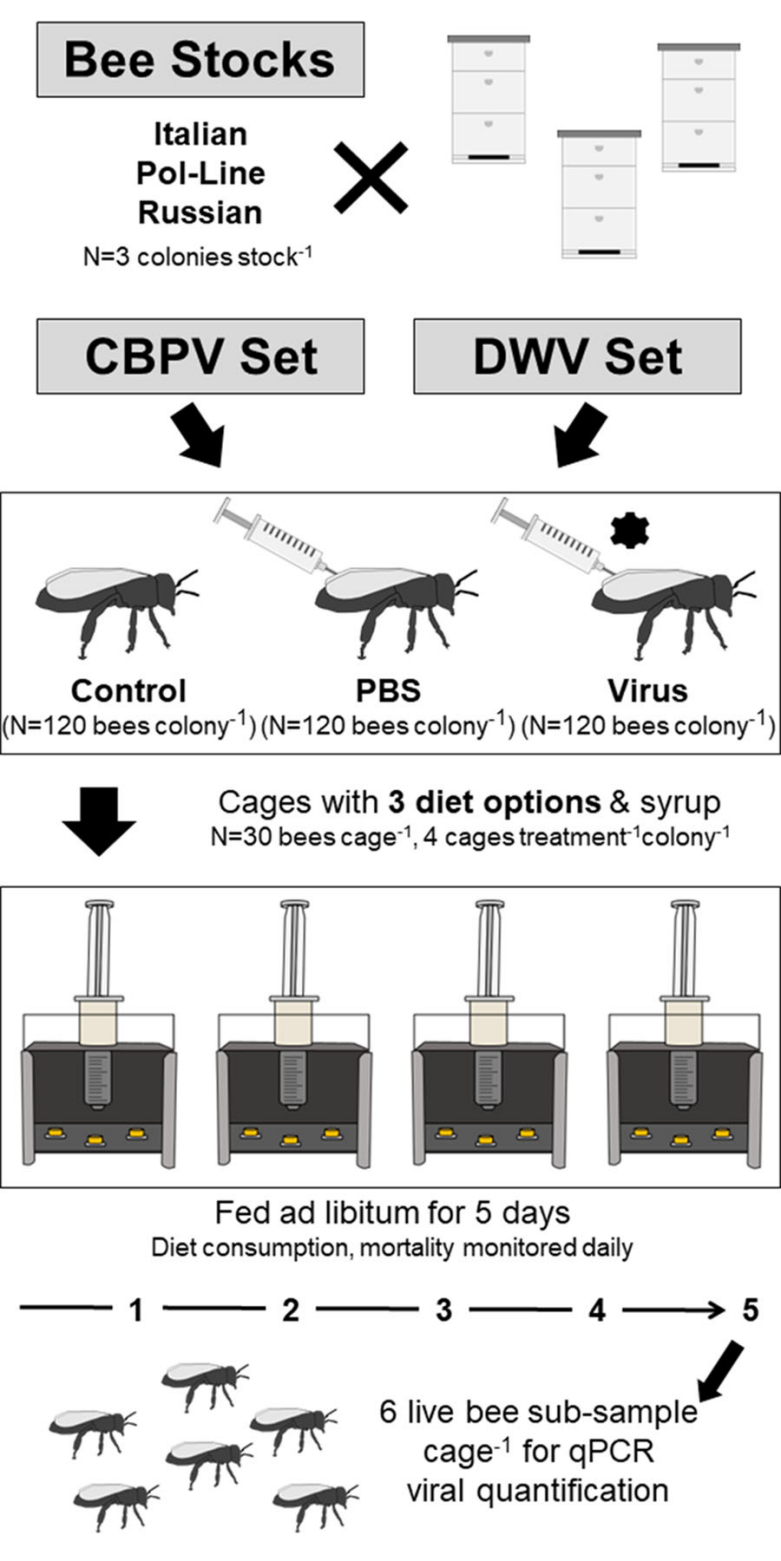
**Experimental design.** Experimental design to determine diet and syrup consumption in relation to bee stock (Italian, Pol-Line, and Russian bees) and treatment (no injection control/naturally occurring infection, PBS sham injection, or sublethal virus injection). The treatments were replicated in their entirety for both CBPV and DWV experimental sets (*N*=4 cages of 30 bees treatment^−1^ colony^−1^ stock^−1^ set^−1^, or *N*=3240 total bees for each experimental set).

The macronutrients of interest were protein and lipids as preferences for these macronutrients are more likely to change with stressors or correlate with preferred pollen compared to carbohydrates (Archer et al., 2014; Vaudo et al., 2016). We investigated two ssRNA viruses [chronic bee paralysis virus (CBPV) or DWV genotype A (DWV-A)] that have different physiological impacts to assess both specific and generalized responses to viral infection (Fig. 1). CBPV has clear, overt effects on neural functioning and is a potential emerging threat in Europe and North America (Budge et al., 2020; Pfeiffer and Crowder, 2022; Ribière et al., 2010; Traynor et al., 2016), while DWV is the most prevalent honey bee virus and causes largely covert or sublethal effects from adult infection (Martin and Brettell, 2019; Traynor et al., 2016). We hypothesized that infection by either virus would result in decreased diet consumption but also an increased preference for protein-enriched diet relative to uninjected controls, but that the extent of this preference would differ among bee stocks. Although being genetically distinct from each other and Italian bees (Saelao et al., 2020), we anticipated that both mite-resistant Pol-Line and Russian stocks will exhibit more similar virus-induced foraging responses to each other than to the susceptible Italian stock.

## RESULTS

### Diet consumption

**Fig. 2.**
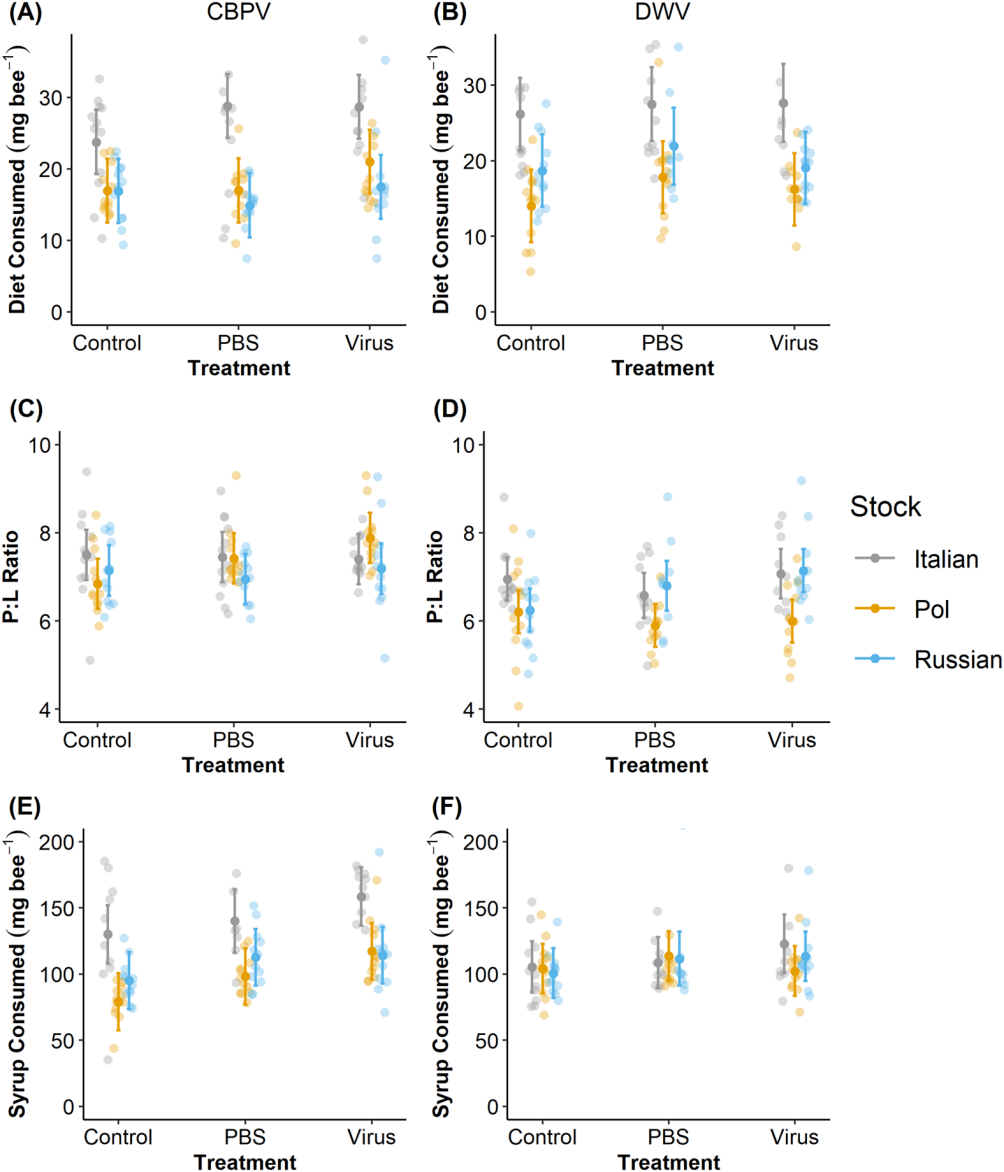
**Diet consumption, diet P:L ratio, and syrup consumption per bee stock and treatment.** A and B depict that Italian bees consumed more diet overall than Russian or Pol-line bees regardless of injection treatment or viral species (see Table 1). C shows that Pol-line bees injected with CBPV ate diet with a higher P:L, while D displays only a that Pol-line bees had a general preference for a lower P:L regardless of treatment in the DWV trial (refer to Table 2). E and F display a general increase in sugar consumption by virus-injected bees and by Italian bees (see Table 3). Center points indicate the associated model-predicted means with standard errors for each bee stock and treatment combination; each point represents one cage; all observations are included (*N*=4 cages of 30 bees treatment^−1^ colony^−1^ stock^−1^ set^−1^, or *N*=108 total cages for each experimental set).

In the CBPV experiment, bee stock was the only marginally nonsignificant variable influencing food consumption (*χ²*=5.942, *P*=0.051, Table 1). Italian bees (23.1±3.1 mg bee^−1^) consumed more diet than Pol-Line (15.0±2.8 mg bee^−1^) or Russian bees (13.2±2.9 mg bee^−1^) (Tukey HSD test: Italian-Pol-Line, *P*=0.019; Italian-Russian, *P=*0.008; Pol-Line-Russian, *P=*0.701; Fig. 2A). However, PBS and virus injection treatments tended, though not significantly, to increase diet consumption (Table 1).

**Table 1.**
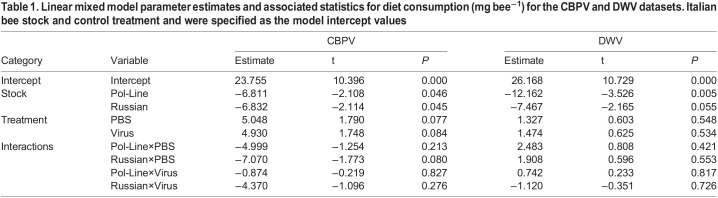
Linear mixed model parameter estimates and associated statistics for diet consumption (mg bee^−1^) for the CBPV and DWV datasets. Italian bee stock and control treatment and were specified as the model intercept values

**Table 2.**
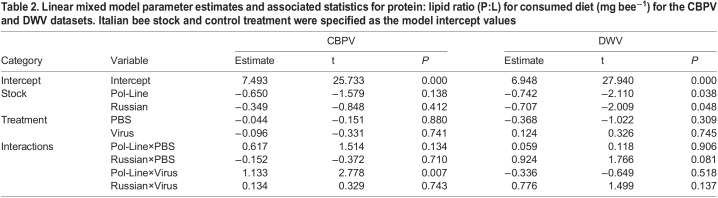
Linear mixed model parameter estimates and associated statistics for protein: lipid ratio (P:L) for consumed diet (mg bee^−1^) for the CBPV and DWV datasets. Italian bee stock and control treatment were specified as the model intercept values

**Table 3.**
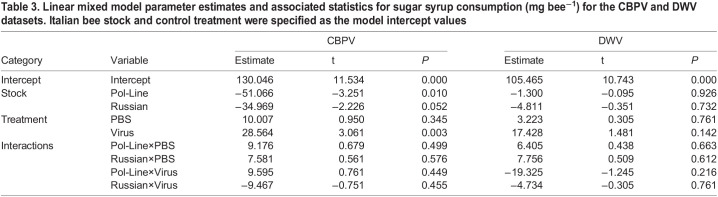
Linear mixed model parameter estimates and associated statistics for sugar syrup consumption (mg bee^−1^) for the CBPV and DWV datasets. Italian bee stock and control treatment were specified as the model intercept values

In the DWV experiment bee stock was the only significant variable (*χ²*=12.647, *P*=0.002, Table 1) influencing food consumption. The Italian bees (26.8±2.3 mg bee^−1^) again consumed significantly more diet than did the Pol-Line bees (15.8±2.3 mg bee^−1^) but not more than Russian bees (19.0±2.3 mg bee^−1^) (Tukey HSD test: Italian-Pol-Line, *P*=0.024; Italian-Russian, *P=*0.116; Pol-Line-Russian, *P=*0.446; Fig. 2B). Note, Russian bees ate less than Italian bees, but this relationship was marginally nonsignificant (*P*=0.055, Table 1).

### Protein: lipid ratio of consumed diet

Protein: lipid ratios of consumed diet in the CBPV experiment were significantly correlated with the bee stock×treatment interaction (*χ²*=9.877, *P*=0.043, Table 2). Bee stock alone (Italian 8.2±0.4:1; Pol-Line: 7.7±0.4:1; Russian: 7.9±0.4:1) was not related to P:L ratios (*χ²*=2.497, *P*=0.287, Table 2). However, Pol-Line bees that were injected with CBPV increased their consumed P:L ratios to a greater extent than Italian bees injected with CBPV (Table 2, Fig. 2C; Fig. S1A).

Unlike the CBPV experiment, P:L ratios in the DWV experiment were impacted by bee stock (*χ²*=5.666, *P*=0.059, Table 2). Italian (6.9±0.2:1) and Russian bees (6.6±0.2:1) consumed diet with higher P:L ratios compared to Pol-Line bees (6.00±0.2:1) (Tukey HSD test: Italian-Pol-Line, *P=*0.020; Italian-Russian, *P=*0.802; Pol-Line-Russian, *P=*0.039; Table 2, Fig. 2D). While no stock×treatment interaction effect was significant, the Russian bees in the PBS treatment did exhibit a marginal increase in consumed P:L ratios compared to Italian bees in the PBS treatment (Table 2, Fig. 2D; Fig. S1B).

### Sugar syrup consumption

In the CBPV experiment, bee stock (*χ²*=10.980, *P*=0.004) and treatment (*χ²*=9.522, *P*=0.009) both significantly impacted sugar syrup consumption (Table 3, Fig. 2E). Italian bees (131.9±11.4 mg bee^−1^) consumed marginally greater quantities of sugar syrup than Russian bees (106.3±10.6 mg bee^−1^) and significantly greater quantities compared to Pol-Line bees (89.3±11.2 mg bee^−1^) (Tukey HSD test: Italian-Pol-Line, *P*=0.042; Italian-Russian, *P=*0.096; Pol-Line-Russian, *P=*0.791). Further, virus injection with CBPV increased syrup consumption relative to control and PBS treated bees (Tukey HSD test: Control-PBS, *P*=0.012; Control-Virus, *P<*0.001; PBS-Control, *P=*0.046; Table 3, Fig. 2E).

The DWV experiment regression indicated that syrup consumption was not impacted by bee stock or treatment (Table 3; Fig. 2F). Similarly, Tukey HSD tests found that all stocks consumed similar quantities of sugar syrup (Italian: 108.4±9.0 mg bee^−1^= Russian: 108.2±8.7 mg bee^−1^= Pol-Line: 106.7±9.2 mg bee^−1^) (Tukey HSD test: Italian-Pol-Line, *P*=0.870; Italian-Russian, *P*=0.938; Pol-Line-Russian, *P=*0.985).

### Virus levels

**Fig. 3.**
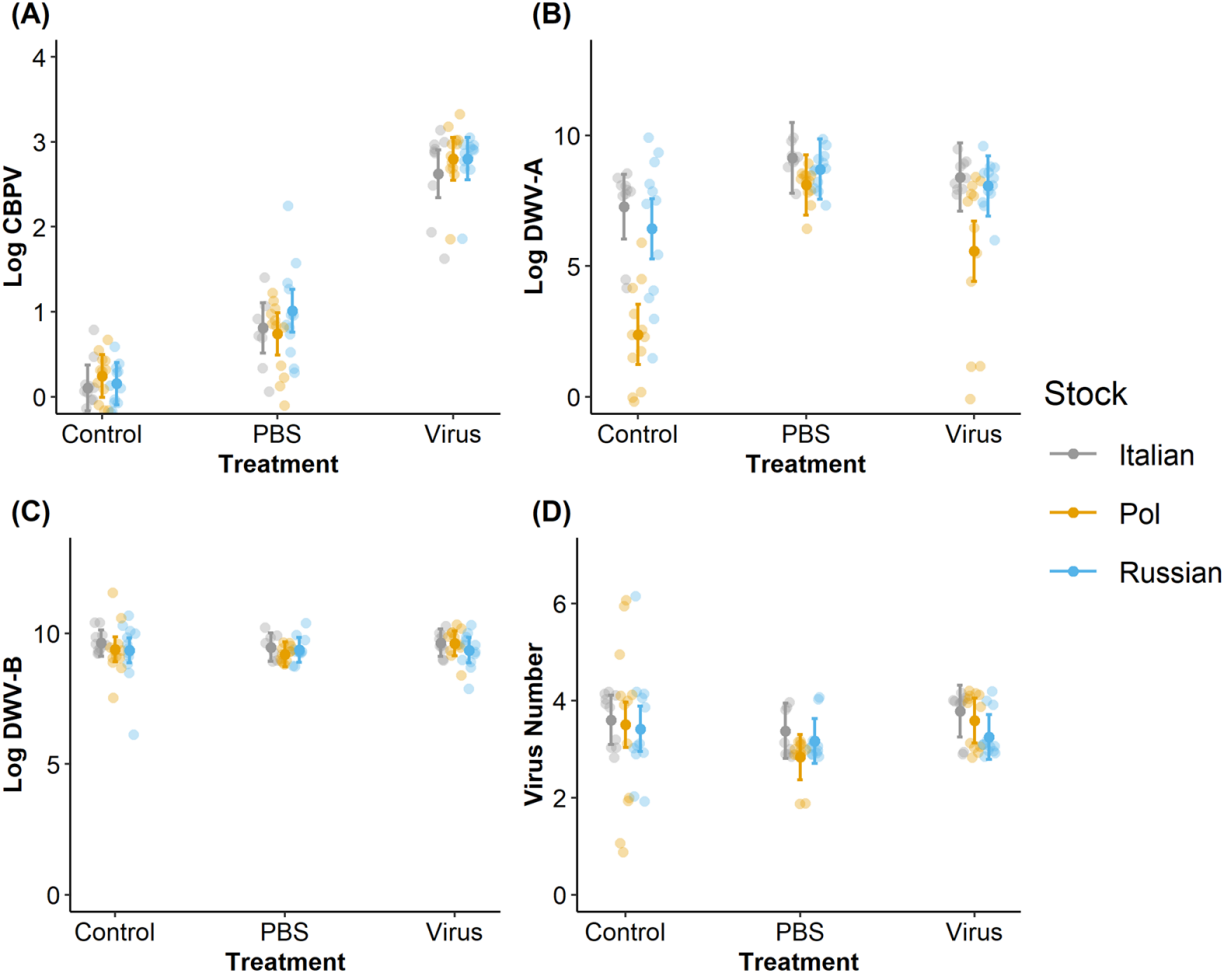
**Comparison of viral titers with treatment in the CBPV experiment.** CBPV titers (A), DWV-A titers (B), DWV-B titers (C), and the total number of viruses (D) found per stock per treatment within the CBPV experimental set. Center points indicate the associated model-predicted means with standard errors for each bee stock and treatment combination; each point represents one cage's six-bee pool; all observations are included (*N*=4 pools of six bees treatment^−1^ colony^−1^ stock^−1^ set^−1^, or *N*=108 total pools for each experimental set).

In the CBPV experiment, we observed that the treatments were effective based on CPBV titers from the pooled bee samples (*χ²*=219.655, *P*<0.001, Fig. 3A). CBPV virus injection treatment significant increased CBPV titers compared to PBS and control treatments (Tukey HSD test: Control-PBS, *P*<0.001; Control-Virus, *P*<0.001; PBS-Control, *P*<0.001). We did not observe stock-based differences in CBPV titers (*χ²*=0.587, *P*=0.746) nor any stock×treatment interactions (*χ²*=3.377, *P*=0.497). However, all viruses except for LSV were also observed at least once (Table S1) and are assumed to have resulted from naturally occurring infections. DWV-A titers (Fig. 3B) (but not DWV-B or virus number, Fig. 3C and D, respectively) exhibited significant stock (*χ²*=38.025, *P*<0.001) and stock×treatment interactions (*χ²*=17.377, *P*=0.002).

**Fig. 4.**
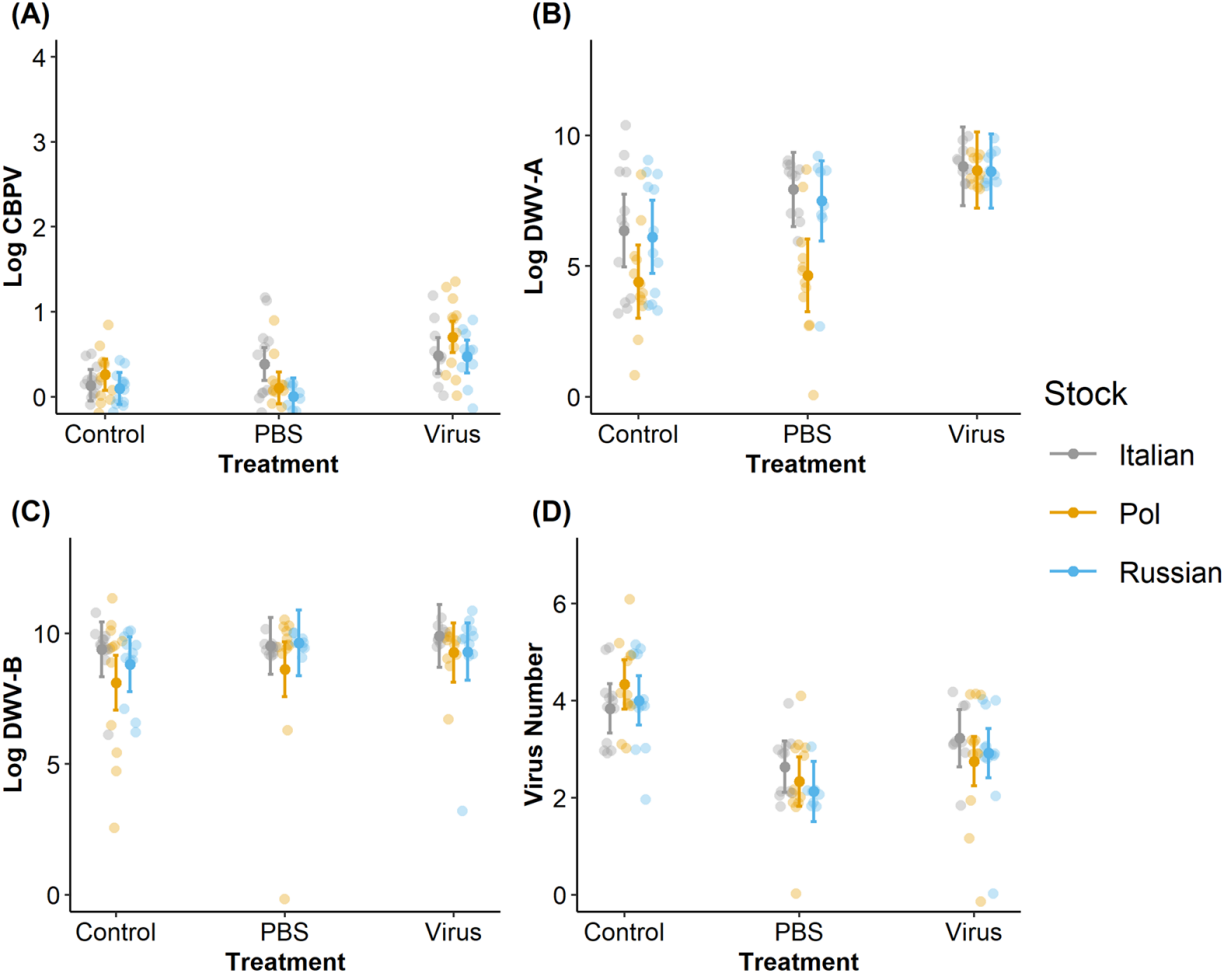
**Comparison of viral titers with treatment in the DWV experiment.** CBPV titers (A), DWV-A titers (B), DWV-B titers (C), and the total number of viruses (D) found per stock per treatment within the DWV experimental set. Center points indicate the associated model-predicted means with standard errors for each bee stock and treatment combination; each point represents one cage's six-bee pool; all observations are included (*N*=4 pools of six bees treatment^−1^ colony^−1^ stock^−1^ set^−1^, or *N*=108 total pools for each experimental set).

In the DWV experiment, we observed that the treatments were effective based on DWV-A titers from the pooled bee samples (*χ²*=12.915, *P*=0.002, Fig. 4B). DWV virus injection treatment significant increased DWV-A titers compared to PBS and control treatments (Tukey HSD test: Control-PBS, *P*=0.021; Control-Virus, *P*<0.001; PBS-Control, *P*<0.001). DWV injections also increased CBPV titers (*χ²*=7.612, *P*=0.022, Fig. 4A), assumed to be from naturally occurring infections (Tukey HSD test: Control-PBS, *P*<0.001; Control-Virus, *P*<0.001; PBS-Control, *P*<0.001). Both DWV-A (*χ²*=12.937, *P*=0.012) and CBPV titers (*χ²*=9.333, *P*=0.053) exhibited interactions between stock and treatment.

We conducted an MCA on data combined from both experimental sets to determine potential treatment associations with the overall virus community other than DWV as most bees tested positive for both types (Table S1, Fig. S2). We found that the presence of naturally occurring viruses was associated with non-injected control bees (Fig. S2).

### Mortality

**Fig. 5.**
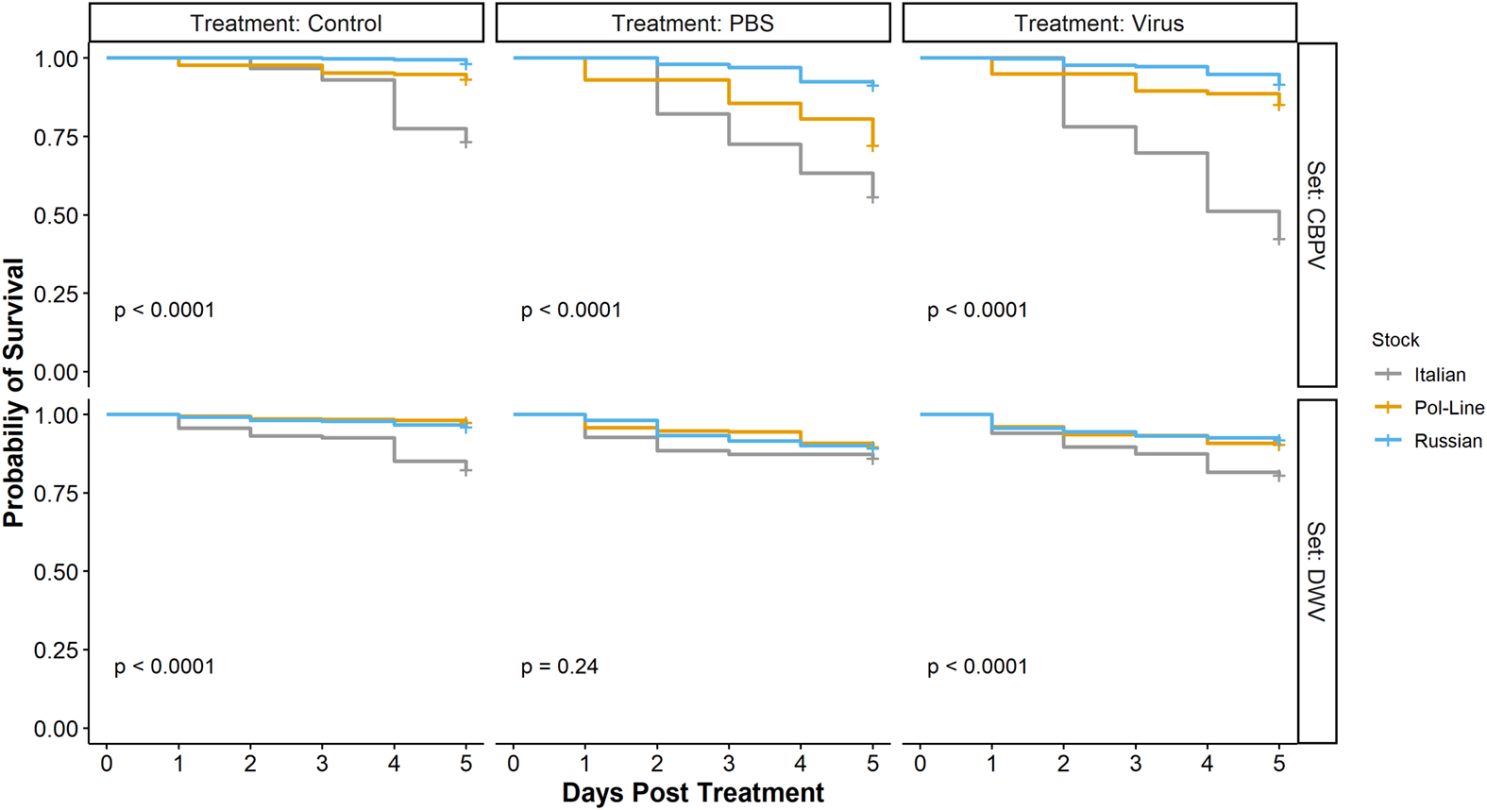
**Kaplan-Meier survival curves comparing bee stocks per treatment in both experimental sets.** Data are represented for the CBPV experiment (top) and DWV experiment (bottom) separately. *P*-values indicate overall statistical differences in survival among bee stocks and within treatment groups for each of the two experimental sets. Each individual bee was included in this analysis (*N*=120 bees treatment^−1^ colony^−1^ stock^−1^ set^−1^, or *N*=3240 bees total for each experimental set).

**Fig. 6.**
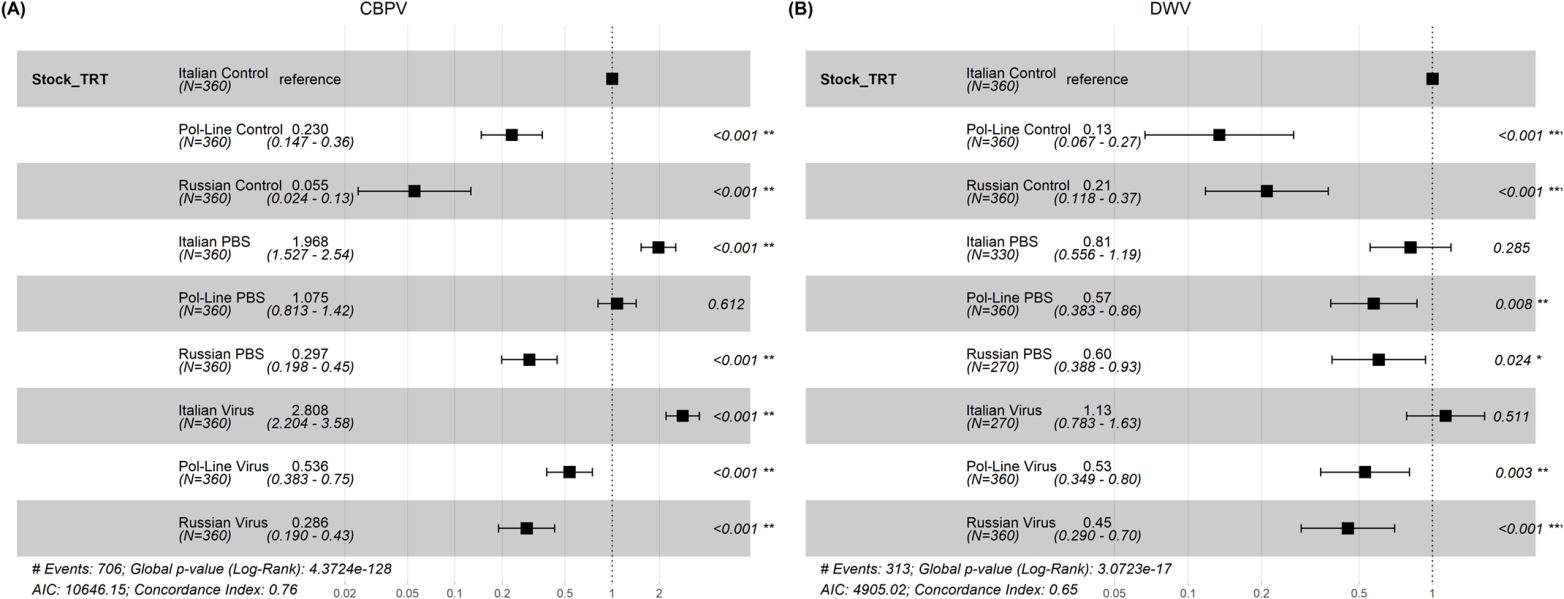
**Hazard ratios of each bee stock×treatment combination in both experimental sets.** Data are represented for the CBPV experiment (A) and DWV experiment (B) separately. The Italian bee stock and control treatment combination was specified as the reference for comparison in both experimental sets. Values less than the reference point, indicate lower likelihood of bee death, whereas values greater than the reference point indicate greater likelihood of bee death relative to the Italian control bees. Sample sizes (N) for each combination are specified in parentheses.

For the CBPV experimental set, the mixed Cox model results indicated that mortality was significantly impacted by bee stock, treatment, and stock×treatment interactions (Table 4, Figs 5A and 6A). Generally, Italian bees had the lowest survival followed by Pol-Line and Russian bees. PBS and CBPV injections had lower survival relative to the control treatment, but the differences between PBS and CBPV differed based on bee stock. When we analyzed the final percentage of dead bees per cage, we found that treatment, syrup consumption, and stock×treatment interactions were significant, reflecting the results of the survival analyses. Both PBS and CBPV injections were positively correlated with mortality (Table 5). Increased syrup consumption was also positively associated with mortality (Table 5; Fig. S3).

**Table 4.**
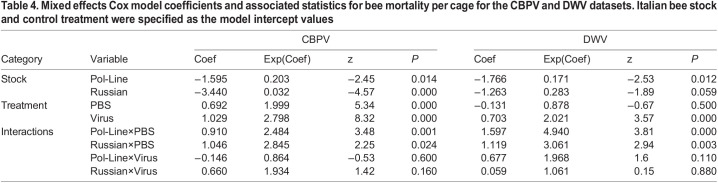
Mixed effects Cox model coefficients and associated statistics for bee mortality per cage for the CBPV and DWV datasets. Italian bee stock and control treatment were specified as the model intercept values

**Table 5.**
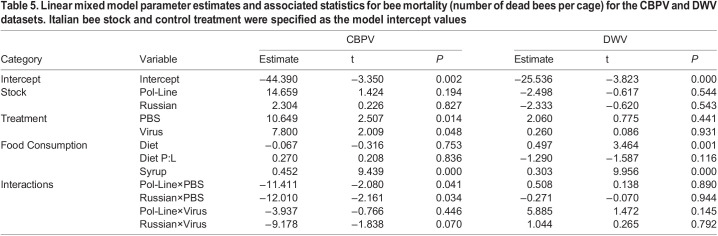
Linear mixed model parameter estimates and associated statistics for bee mortality (number of dead bees per cage) for the CBPV and DWV datasets. Italian bee stock and control treatment were specified as the model intercept values

For the DWV experimental set, the mixed Cox model results again indicated that mortality was significantly impacted by bee stock, treatment, and stock×treatment interactions (Table 4, Figs 5B and 6B). Like the CBPV experimental set, Italian bees in the DWV set had the lowest survival followed by Pol-Line and Russian bees. DWV injections had lower survival relative to the control treatment, but the differences were based on bee stock. When we analyzed the final percentage of dead bees per cage (Table 5), we found that increased diet and syrup consumption were positively associated with mortality (Table 5; Fig. S4).

## DISCUSSION

### Virus identity and titer on foraging

One component of this study was to determine if viral infection alters adult bee dietary preferences and if similar responses would be seen for two ssRNA viruses (DWV and CBPV) since ample empirical evidence shows that microbial infection reduces feeding in various insects (Hsu et al., 2018; Mason et al., 2014; Povey et al., 2014). We expected that injections of both viruses would decrease bees' overall diet consumption (Adamo et al., 2010), but increase preferences for protein (DeGrandi-Hoffman et al., 2010; Dolezal et al., 2019). We found that changes in total diet consumption and sugar syrup consumption varied with virus species. CBPV increased syrup consumption as previously shown with *N. ceranae* (Naug and Gibbs, 2009); conversely, DWV injection did not alter either diet or syrup consumption. The differences in syrup consumption associated with the two viruses may be due to their relative impacts on honey bee physiology and associated metabolic demands (Iqbal and Mueller, 2007; Wang et al., 2010). For instance, *N. ceranae* and *N. apis* have both been found to impose energetic costs on honey bees, but *N. ceranae* to a much greater extent than *N. apis* (Kurze et al., 2016; Martín-Hernández et al., 2011). DWV and CBPV cause divergent symptomologies and likely physiological responses (Budge et al., 2020; Martin and Brettell, 2019; Ribière et al., 2010; Traynor et al., 2016), which may, in turn, require different nutritional needs and induce differential foraging or feeding behaviors.

Changes in foraging can be beneficial to the forager in terms of either reduced pathogen loads or increased survival but may depend on the pathogen identity or susceptibility of the infected host (Annoscia et al., 2017; Foley et al., 2012; Genersch et al., 2005; Jack et al., 2016; McMahon et al., 2016). Overall, cages with higher mortality consumed a greater quantity of both diet and sugar syrup (Figs S3 and S4), indicating that the potential physiological responses to viruses and injury are energetically costly (Aldea and Bozinovic, 2020; Ardia et al., 2012). The importance of these interactions is potentially due to the differential immune responses to pathogens and injury by different stocks (Evans and Pettis, 2005) or differential tolerance of stocks to viruses (Khongphinitbunjong et al., 2016; Locke et al., 2014; Penn et al., 2021). Given that we studied sub-lethal virus doses, our study was not set up to determine if virus-induced feeding patterns prevent mortality; therefore, further study into this aspect is necessary. Additionally, how this change in feeding patterns in the cage setting would translate into potential alteration in foraging differences or utilization of stored hive resources needs to be investigated more fully in terms of how it would ultimately impact colony health and survival.

### Importance of bee genotype

The second goal of this study was to determine if honey bee genotypes, particularly those bred for *Varroa* mite resistance (Pol-Line and Russian), differed in their foraging behaviors relative to susceptible Italian bees after exposure to viruses as suggested from differences in diet choice. Our hypothesis that there would be genotypic differences was generally supported. Italian bees consumed more diet than either the Russian or Pol-Line bees during both experimental sets, which is not surprising given the known frugality of Russian bees and the documented consumption patterns of Italian bees (de Guzman et al., 2005; DeGrandi-Hoffman et al., 2021). There may have also been lasting impacts from source colony *Varroa* infestations, reflecting genotypic differences in mite resistance. For instance, the consumption patterns of Italian bees in our study reflect those of a prior study showing that regardless of viral loads, bees from *Varroa*-infested colonies consume more pollen (Annoscia et al., 2017). However, bee genotype responses did not entirely align with *Varroa* mite resistance (Italians versus Pol-Line and Russian). Pol-Line bees consumed lower P:L ratios relative to both Italian and Russian bees in the DWV experiment

Further, P:L ratios of consumed diet were impacted by bee genotype×treatment interactions (Kurze et al., 2016; Page et al., 1998). We found that CBPV injection increased P:L as expected in Pol-Line bees (Erler and Moritz, 2016; Povey et al., 2014). While DWV injection did not alter P:L ratios, PBS injection within the DWV experimental set marginally increased the consumed P:L ratios of Russian bees. This interaction of PBS injection rather than DWV injection inducing a change in Russian bee P:L preferences in the DWV experimental set might be due to the combination of injection trauma and naturally occurring viral infections (Figs 3 and 4). Further, PBS injections in the CBPV experimental set increased syrup consumption, indicating that bodily injury alone may induce changes in feeding behavior (Chen et al., 2006b; Möckel et al., 2011; Ribière et al., 2007). Control bees were also more likely to be infected with other viruses such as ABPV, BQCV, IAPV, and KBV, particularly within the DWV experiment (Fig. S2). This may indicate that the stress response induced by injections may help clear these infections (Browne et al., 2014; Sheehan et al., 2020) or that injection trauma may facilitate displacement by inducing replication of competing viruses (Carrillo-Tripp et al., 2016; Remnant et al., 2019; Shen et al., 2005).

Bee genotype differences in relation to different viruses are expected as colony-level tolerance/resistance to viruses like DWV, BQCV, and Sacbrood virus have previously been documented in *Varroa*-resistant colonies (Khongphinitbunjong et al., 2016; Locke et al., 2014; Thaduri et al., 2019; Weaver et al., 2021). In this study, the bee genotype×virus interactions were not always consistent for the two resistant stocks relative to the susceptible stock or between viral species. While both Italian and Russian bees had higher levels of DWV-A than Pol-Line, Italian bees experienced the highest levels of mortality while Russian bees had the lowest of the three bee genotypes (indicating potential DWV-A tolerance) (Penn et al., 2022a). Taken together, consumption patterns, viral loads, and mortality indicate that the two mite-resistant genotypes - Pol-Line and Russian - are not entirely similar to each other, reflecting underlying genomic differences (Saelao et al., 2020) such as metabolic rate or nutrient conversion efficiency (DeGrandi-Hoffman et al., 2021; Ricigliano et al., 2021).

### Future considerations

Our study indicates that individual bees are potentially capable of changing their feeding and possibly foraging preferences based on their infection status and that this varies among bee genotypes and with virus species. Given that nurse and worker bees may have different viral loads (Chen et al., 2006a) and ability to discriminate pollen/preferences based on macronutrients from foragers (Corby-Harris et al., 2018; Paoli et al., 2014; Stabler et al., 2021), we also need to consider forager macronutrient preferences and if they are based on individual or colony health (Katz and Naug, 2016; Lihoreau et al., 2015; Penn et al., 2022b; Poissonnier et al., 2017). Viruses like DWV also induce precocious behavioral maturation within affected colonies (Benaets et al., 2017; Traniello et al., 2020) and decrease the effectiveness of foragers by reducing flight distances and duration as well as homing rates (Iqbal and Mueller, 2007; Wells et al., 2016). Diminished foraging capacity and efficiency can then contribute to colony loss (Perry et al., 2015). Bee genotypes exhibit inherent foraging differences potentially due to the different colony demands like brood rearing and food storage that may further interact with parasite and pathogen stressors (Danka et al., 1987; Fewell and Page, 2000; Guzman-Novoa and Gary, 1993; Pankiw and Page, 2001; Pankiw and Page, 1999). Given the observed interactions of two viruses with bee genotype on foraging in this cage study, field evaluation of individual and colony foraging combined with mite presence and virus loads across bee genotypes is necessary to better understand the ramifications of these data for colony health (DeGrandi-Hoffman et al., 2020; Dubois et al., 2018; Penn et al., 2022b).

Our results show that the species of viral infection coupled with honey bee genotype can result in differential diet and sugar syrup consumption rates and macronutrient preferences. We found that for the two ssRNA viruses tested, one that covertly influences lifespan and behavior (DWV-A) and one that causes overt mortality and neurological paralysis (CBPV), as well as the injection damage itself as indicated by PBS treatments, had differential interactions. We also observed that Italian bees may benefit from additional food supplementation more so than mite-resistant stocks, especially since they are also more susceptible to virus-vectoring *Varroa* mites (however, see DeGrandi-Hoffman et al., 2020). Although Russian bees had higher DWV-A levels than Pol-Line, on par with Italian bees, they exhibited lower mortality. These data may indicate competing demands for nutritional resources when bees encounter different viruses or differences in how bee stocks are able to adapt given different viral versus physical stressors. Future work needs to be done to determine the mechanistic differences among bee genotypes and viruses that induce these changes in nutritional preferences and if colony longevity is influenced by these decisions.

## MATERIALS AND METHODS

### Diet preparation

To evaluate bee macronutrient preferences, we created three pollen-substitute diets varying in protein: lipid ratios. The protein and lipid contents for the powdered base (Ultra Bee Dry Feed, Mann Lake LTD, Hackensack, MN, USA), bovine casein (protein addition, MP Biomedicals, Irvine, CA, USA), and soybean lecithin (lipid addition, Beantown Chemical Corporation, Hudson, NH, USA) were determined using a Bradford assay and chloroform-methanol extraction, respectively (Conte et al., 2017; Stabler et al., 2021; Vaudo et al., 2016). For protein analyses, three samples (∼200 mg each) of each diet component were pre-weighed then dissolved in 700 µl of TE buffer. Samples were homogenized using a handheld pestle and vortexed. Ten µl of the mixed samples were diluted in 190 µl of deionized water and briefly vortexed. Standards (0, 0.04, 0.08, 0.12, 0.16, 0.20, 0.24, and 0.28 mg ml^−1^) were created using BSA (Bio-Rad Laboratories, Hercules, CA, USA). Five µl of each sample and standard in triplicate were combined with 245 µl of Bradford reagent (Bio-Rad Laboratories, Hercules, CA, USA), incubated for 5 min, and analyzed using a spectrophotometer (SpectraMax Plus Microplate Reader, Molecular Devices, San Jose, CA, USA) at 595 λ. For lipid content analysis (Bligh and Dyer, 1959; Manirakiza et al., 2001; Vaudo et al., 2016), three samples (200 mg each) of each diet component were dried in a desiccator at room temperature for 24 h, pre-weighed then vortexed with 0.2 ml 2% sodium sulfate. One ml of chloroform-methanol (2:1) was then added, vortexed, then centrifuged 2180 G for 5 min at room temperature. 300 µl of the precipitated layer was combined with 0.6 ml of deionized water, vortexed, centrifuged again, and incubated at 90°C for 20 min. 300 µl of sulfuric acid (Thermo Fisher Scientific Inc., Waltham, MA, USA) was added then samples incubated at 90°C for 20 min followed by a 2 min ice bath for samples to reach room temperature. One hundred μl of each sample was read on the spectrophotometer at 540 λ. Samples were compared against canola oil standards (0, 0.015, 0.03, 0.06, 0.09, 0.12, 0.15, and 0.18 mg ml^−1^).

Based on the protein and lipid composition of the components, diets were formulated so that all bees were presented with a range of options: standard diet (P:L of 8.33:1), a standard+protein option (P:L of 10.58:1), and a standard+lipid option (P:L of 2.431:1). The standard diet consisted of 20 g Ultra Bee Dry Feed (Mann Lake LTD, Hackensack, MN, USA), 35 ml 50% sucrose solution, and 5 ml 50% glycerol. For the treatment diets, 17.39 g Ultra Bee Dry Feed was combined with either 2.61 g bovine casein (protein-addition) or soybean lecithin (lipid-addition), 35 ml 50% sucrose solution, and 5 ml 50% glycerol (Ricigliano and Simone-Finstrom, 2020). Individual diet units were created by filling the caps of autoclaved 1.5 ml microcentrifuge tubes to the rim (∼0.5 g). Diet was prepared in batches of approximately 100 feeding units. All diet units were stored in Ziplock bags at −20°C until use when they were thawed to room temperature for initial weighing.

### Viral isolation for inoculum

To obtain viral inoculum for injections, a group of ten symptomatic CBPV bees and ten adult bees with phenotypic DWV traits were separately frozen at −80°C, ground to a fine powder, homogenized in 10 ml sterile 1X PBS, and centrifuged at 5000 rpm at 4°C for 20 min. The supernatants containing the viruses were manually filtered through a 0.2-micron filter (milex-GS syringe filter unit #SLGS033SS, Millipore Sigma, Burlington, MA, USA) to remove small tissue debris, fungi, and bacteria. qPCR was conducted to test for the presence of non-target viruses using methods described below. Viral quantification for CBPV and DWV was performed by absolute quantification using the Standard Curve Method. All methods were previously established based on standard protocols (Simone-Finstrom et al., 2018). One sample stock solution each for CBPV or DWV was selected based on negative results for non-target viruses and used to create the injection stock solution. CBPV stocks were diluted to 10^2^ while DWV stock solutions were diluted to 10^5^ in sterile 1X PBS, doses previously established as biologically relevant but sublethal to adult bees (Gisder et al., 2009; Mookhploy et al., 2021).

### Feeding behavior bioassay

See Fig. 1 for graphical representation of method and sample sizes. Newly emerged adult bees (<24 h old) were collected from three colonies of each honey bee stock (Italian, Pol-Line, and Russian) then randomly assigned to one of three treatment groups (*N*=120 bees treatment^−1^ colony^−1^ experimental set^−1^): (a) no injection control, )b) sterile 1X PBS injection (3.0 µl), or c) virus injection (CBPV or DWV) (3.0 µl). The DWV experiment evaluated the effect of DWV on macronutrient intake and was conducted from 25 June to 3 July 2019, while the CBPV experiment was conducted from 9 to 16 July 2019. Each experiment included its own set of control and PBS-injected bees in addition to the experiment-specific virus-injected bees. To facilitate injections, bees (including no injection controls) were placed in scintillation vials on ice for 2 min to slow movements. PBS and virus treatments were then injected using a 30G needle (Hamilton Company, Reno, NV, USA) inserted into the lateral abdomen between the fourth and fifth pleurites, based on established protocols (Simone-Finstrom et al., 2018). An UltraMicroPump with a SYS-Micro4 Controller (World Precision Instruments, Sarasota, FL, USA) with an infusion flow rate of 1.0 µl s^−1^ was used, following the manufacturer's parameters.

Following injections, bees were housed in cages (*N*=30 bees of same treatment and colony cage^−1^, for a total of four cages treatment^−1^ colony^−1^ experimental set^−1^) and kept in a dark incubator at 34°C with 85% relative humidity (Williams et al., 2013). Each cage was provisioned with one unit (∼0.5 g) of each diet type (standard diet, standard diet+protein, or standard diet+lipid) and 5.0 ml of 50% sucrose (sugar) syrup (Azzouz-Olden et al., 2018; Corby-Harris et al., 2018; Pirk et al., 2010; Raubenheimer and Jones, 2006). Bees were fed *ad libitum* for 5 days with the same diet amount (∼0.5 g unit per diet type) replaced daily. To estimate consumption, the diet units were reweighed daily, while the final sugar syrup weights were collected at the end of the 5 days. To account for diet weight changes due to evaporation, no-bee control cages were established in the same environmental chamber with diet units changed and weighed daily and sugar syrup measured after 5 days. To determine bee mortality, dead bees were removed from each cage and counted daily. At the end of the observation period, all remaining live bees were frozen in pools by cage and stored at −80°C until RNA extraction for viral analyses.

### Viral analyses

Pools of six bees collected on the final day of the study were randomly selected from each cage (*N*=209). After removal of abdomens, heads and thoraces were placed in 2 ml homogenization vials pre-fitted with 1.4 mm ceramic beads (Omni International, Kennesaw, GA, USA), to which 400 µl Promega Homogenization Solution at 5°C (Promega Corporation, Madison, WI, USA) was also added. Following homogenization, 400 µl Promega Lysis buffer was added to each tube and vortexed for 15 s. All samples were centrifuged for 10 min at 4°C at 14,000 rpm. Total sample RNA was extracted from 400 µl cleared lysate using the Maxwell RSC 48 simplyRNA tissue extraction kits and program (Promega Corporation, Madison, WI, USA) according to standard procedures. RNA was stored in 0.6 ml elution tubes wrapped in parafilm (Bemis Company Inc., Oshkosh, WI, USA) at −80°C until cDNA synthesis.

Frozen RNA samples were thawed on 5°C metal beads, vortexed briefly, and centrifuged. Each RNA sample was Nano dropped (NanoDrop One, Thermo Fisher Scientific Inc., Waltham, MA, USA) twice using 1 μl of sample each time. The mean ng µl^−1^ NanoDrop One readings were calculated per sample and used to determine the volume of RNA template and nuclease-free water required to reach a sample concentration of 100 ng RNA. cDNA was synthesized in two steps using Qiagen QuantiTect Reverse Transcription kits (Thermo Fisher Scientific Inc., Waltham, MA, USA). For step one, 2 µl of gDNA wipeout was added to the mix of RNA and water for a total reaction volume of 14 µl per sample. Samples were incubated at 42°C for 2 min in a Bio-Rad T100 Thermal Cycler (Bio-Rad Laboratories, Hercules, CA, USA), briefly vortexed, and centrifuged before the addition of Step 2 reverse transcription master mix comprised of 4 µl 5X Buffer, 1 µl of RT Primer mix, and 1 µl of RT enzyme per sample. Samples were again vortexed and centrifuged and placed into the Bio-Rad T100 Thermal Cycler (42°C for 25 min then 95°C for 3 min). cDNA was stored in strip tubes wrapped in parafilm at −20°C until RT-PCR.

For viral analyses, each pool of bees was analyzed for the following eight viruses: ABPV (Acute Bee Paralysis Virus), BQCV (Black Queen Cell Virus), CBPV, DWV-A, DWV-B (Deformed Wing Virus genotype B), IAPV, KBV (Kashmir Bee Virus), and LSV (Lake Sinai Virus), following established protocols (de Guzman et al., 2017; Pirk et al., 2013; Simone-Finstrom et al., 2018). The reference gene β-actin was used to ensure sample quality (Lourenço et al., 2008). Each sample was replicated three times per primer pair for RT-PCR analyses. All RT-PCRs consisted of 5 µl SsoFast Universal SYBR Green supermix (Bio-Rad, Hercules, CA, USA), 3 µl nuclease-free water, 0.5 µl forward primer, 0.5 µl reverse primer, and 1 µl cDNA template. Reactions were run in Bio-Rad CFX Connect platform (Bio-Rad, Hercules, CA, USA) with all reactions of a specific primer occurred in the same machine. The PCR thermal protocol for the DWV-A and CBPV primer pairs includes a Taq activation step of 95°C for 5 min followed by 40 cycles of 95°C for 5 s, and 53.5°C for 10 s then 72°C for 10 s; while the protocol for ABPV, β-actin, BQCV, DWV-B, KBV, and LSV was Taq activation at 95°C for 5 min followed by 40 cycles of 95°C for 5 s, and 52.5°C for 10 s then 72°C for 10 s. The PCR cycling protocol for the IAPV primer pairs was 95°C for 5 min followed by 40 cycles of 95°C for 5 s and 53.5°C for 10 s then 72°C for 10 s. All thermal protocols included a melt-curve dissociation analysis to confirm product size. CBPV, DWV-A, and DWV-B titers were quantified using the Standard Curve Method using linearized plasmid constructs. All other viruses were counted as positive for any Ct value registered at less than 40 cycles. Three samples from the DWV experiment and nine samples from the CBPV experiment did not test positive for β-actin; therefore, these data points did not include virus data in later analyses.

### Statistical analyses

Diet controls (from bee-free cages) were used to determine weight change due to environmental conditions, which was calculated by subtracting the final weight (after 24 h) from the initial weight (Martín-Hernández et al., 2011; Nicolson et al., 2018). The difference in control amount (corresponding to each diet type and date of the study) was subtracted from the differences in the final and initial weights of each food in cages with bees to give the total amount consumed for each diet cage^−1^ day^−1^. The exception to timing was a single day (13 July 2019) that was skipped during the CBPV experiment due to laboratory closure for a hurricane, which was accounted for by dividing the weight differences by 2 days. The total amount of diet consumed (mg) was then standardized (mg bee^−1^) using the number of bees alive each day. Diet consumption was combined for all days of the experiment to obtain the total consumption bee^−1^. Protein and lipid consumptions were determined by multiplying the final consumption bee^−1^ by the relative concentrations of protein and lipid determined for each diet type during the initial nutritional analyses. Sugar syrup consumption (mg bee^−1^) was calculated for each entire experimental period after taking the environmental controls into account as above.

All analyses were completed in R v4.0.3 (R Core Team, 2022) with all data from the DWV and CBPV experiments analyzed separately. Cages where mortality was greater than 20 of 30 bees during the first two days after injection were censored (*N*=9 for the DWV experiment). Linear mixed models (lmer function from lme4 package) were used to determine if bee stock and treatment (and the interaction thereof) influenced diet and syrup consumption as well as the P:L ratios (Bates et al., 2015). All mixed models used colony as a random factor. *P*-values were estimated using Satterthwaite's method in the lmerTest package (Kuznetsova et al., 2017). Post-hoc Tukey comparisons were conducted with the emmeans package (Lenth, 2020). Bee mortality was analyzed using Kaplan-Meier survival curves and mixed effects Cox models (survival and coxme packages), using the same random effects as above (Therneau, 2022 a; Therneau, 2022 b; Therneau and Grambsch, 2000). The final percentage of dead bees per cage was also analyzed in relation to stock, treatment, and feeding metrics using linear mixed models as above in order to pair foraging data with mortality. Further association of the entire virus community (presence/absence data) with treatments was conducted using a multiple correspondence analysis (MCA) with the FactoMineR package in R (Husson et al., 2008). DWV genotypes A and B were excluded from the MCA as all bees tested positive for both genotypes. All figures were created using ggplot2 (Wickam, 2016).

## Supplementary Material

10.1242/biolopen.059039_sup1 Supplementary information
