# Supplementary material for: Viral species differentially influence macronutrient preferences based on honey bee genotype
Source: Biol Open. 2022 Sep 30;11(10):bio059039. doi: 10.1242/bio.059039 (PMC9548382; doi:10.1242/bio.059039)
Supplement: Supplementary information [file biolopen-11-059039-s1.pdf]

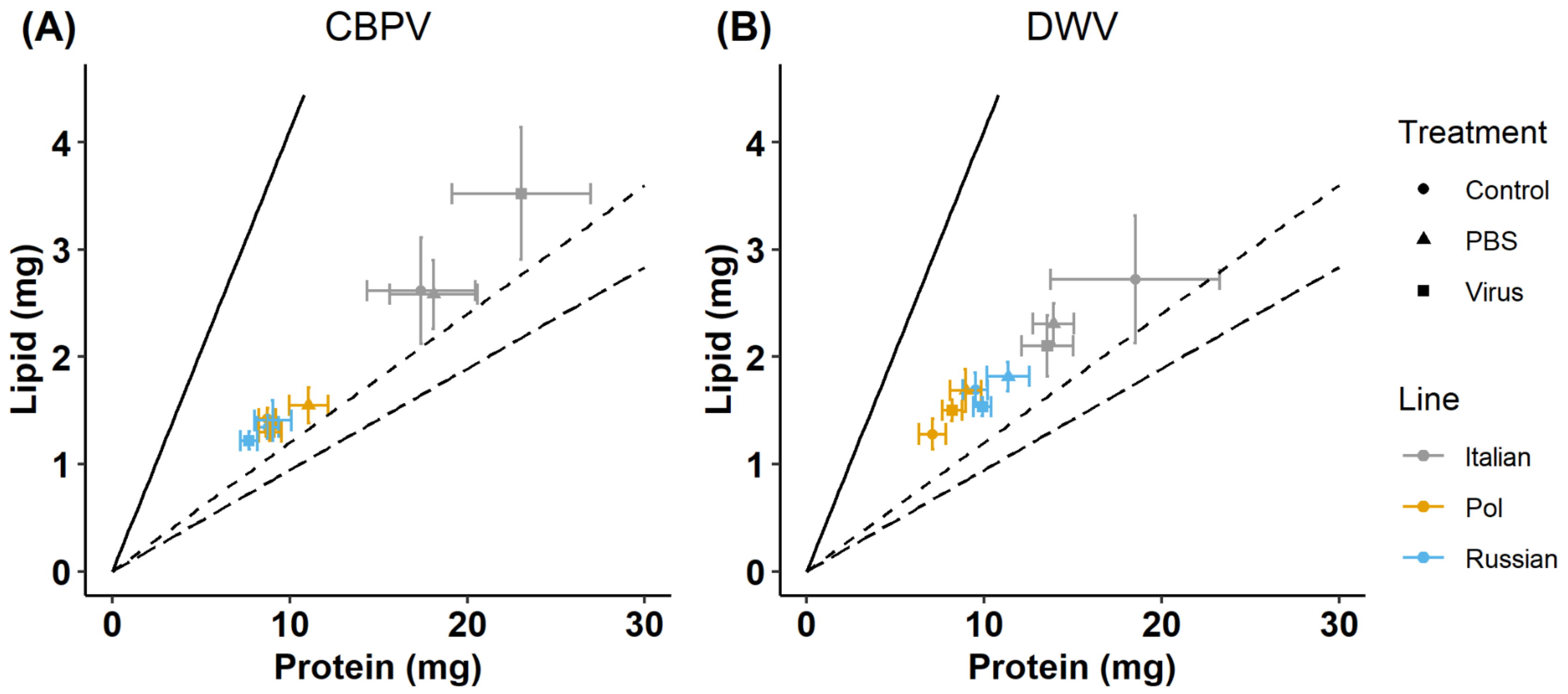

Fig. S1. Protein to Lipid ratios of consumed diet for each bee stock and treatment. Data are represented for the CBPV experiment (A) and DWV experiment (B) separately. Available diet values are represented by the small, dashed line (standard UltraBee diet), the solid line (lipid addition diet); and the large, dashed line (protein addition diet). Points represent the means for each bee stock and injection treatment combination. Standard errors were calculated using cage-level data; all observations are included.

Biology Open • Supplementary information

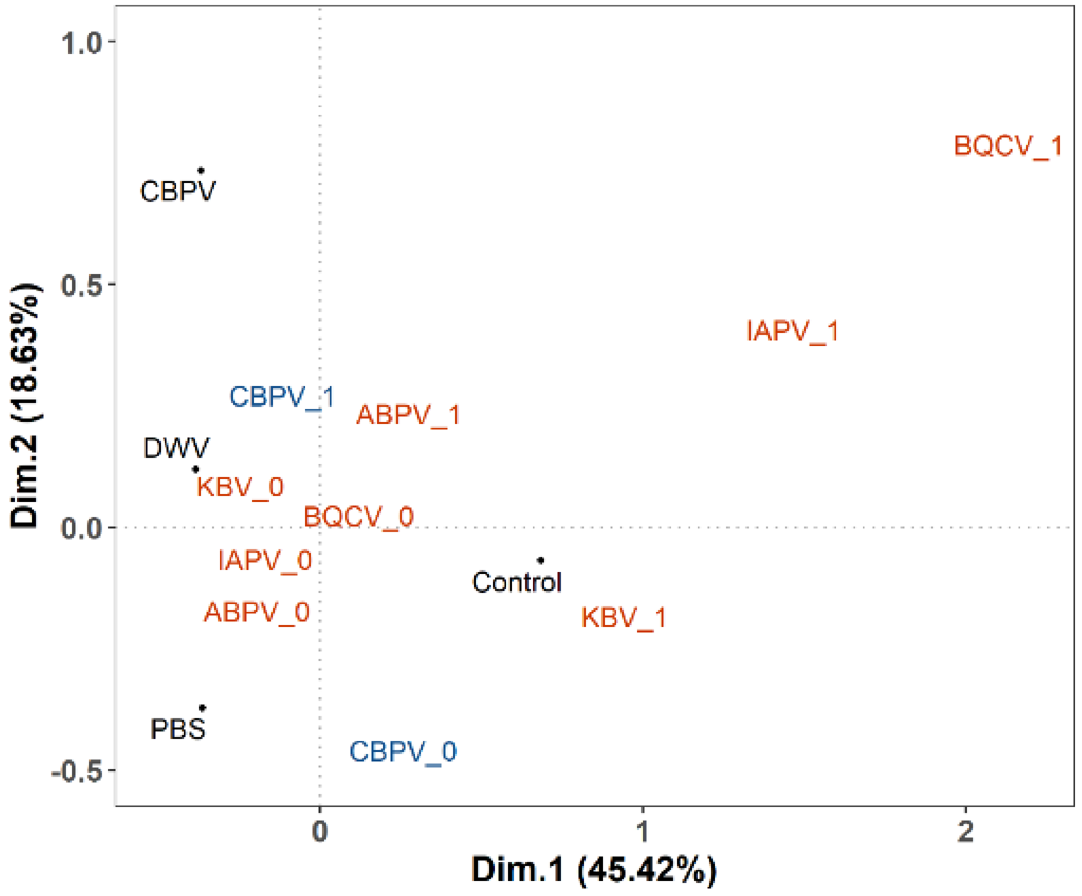

Fig. S2. Multiple correspondence analysis on the combined datasets to evaluate the relationship between the presence (1) and absence (0) of all tested viruses with treatment (Control, PBS-injected, DWV-injected, and CBPV-injected). Eigenvalues are presented for each dimension in parentheses. Dim.1 appears to represent the variation of injected versus non-injected bees, whereas Dim.2 represents the variation between the two viruses (CBPV and DWV) and two control treatments (non-injected and PBS). Since the absence of DWV was rare (genotype A 8/199 instances, genotype B 4/199 instances) as was LSV (1/199 instances), these viruses were excluded from analysis.

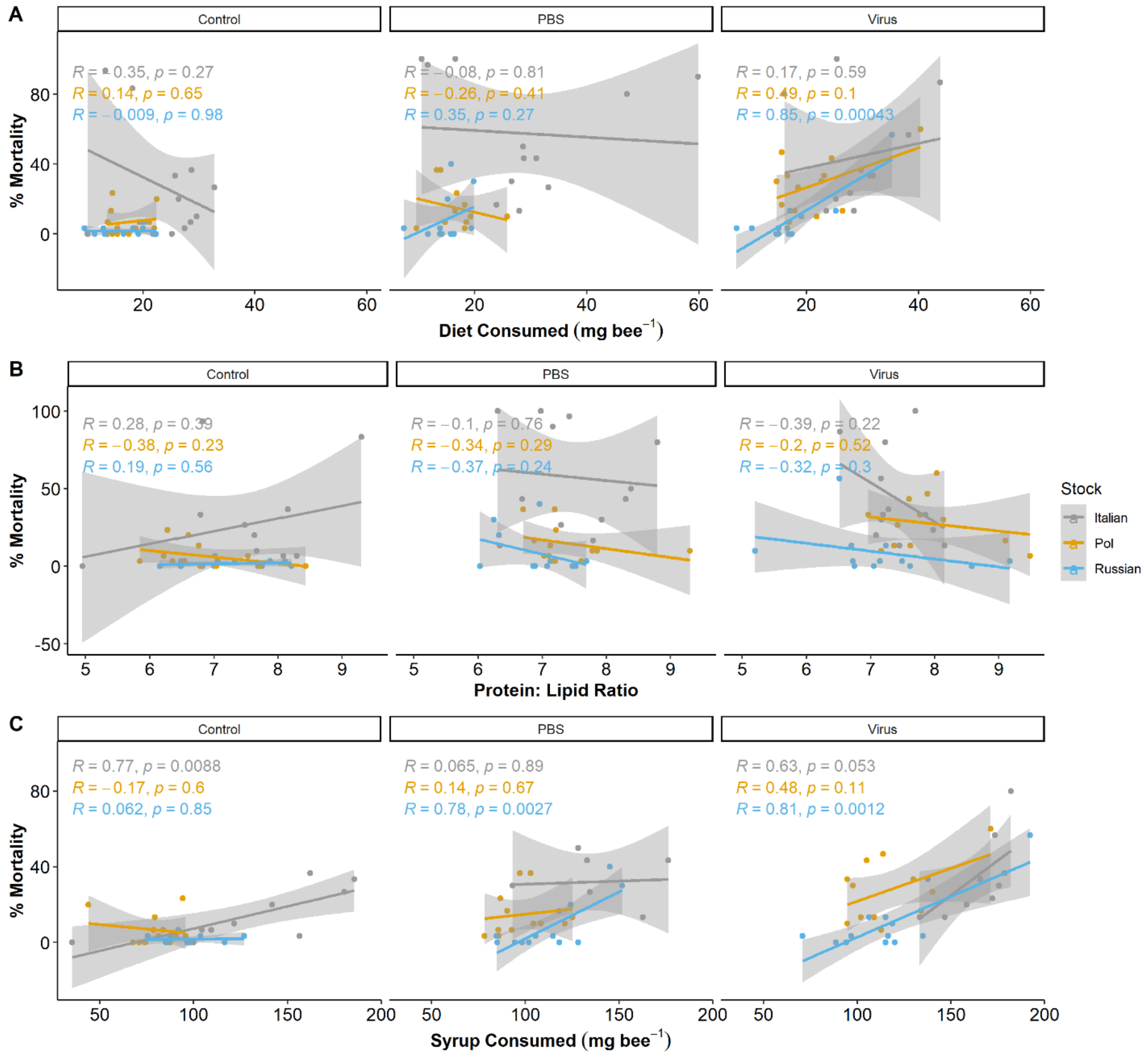

Fig. S3. Final percentage of dead bees per cage in the CBPV experimental set relative to consumed diet, P:L ratio of consumed diet, and consumed syrup. Shading represents the standard error for each line; each point represents one cage, and all observations are included.

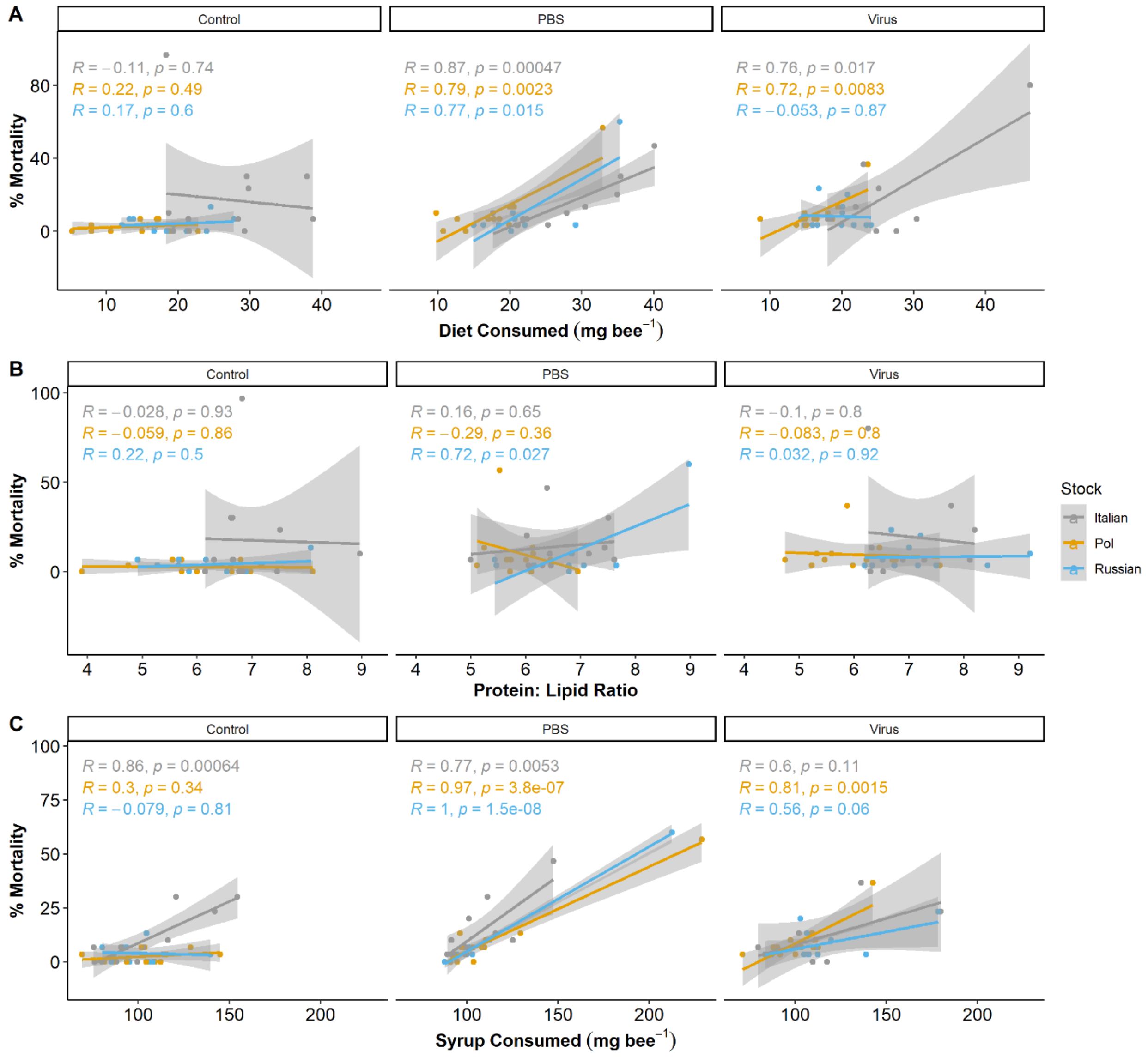

Fig. S4. Final percentage of dead bees per cage in the DWV experimental set relative to consumed diet, P:L ratio of consumed diet, and consumed syrup. Shading represents the standard error for each line; each point represents one cage, and all observations are included.

Table S1. The presence of all tested viruses (Ct values < 40 cycles) for each pooled caged bee sample (N = 4 cages $colony^{-1}$ $stock^{-1}$ $treatment^{-1}$). Missing cages were due to all bees dying before day 5 or extracted RNA sample being of poor quality as indicated by β-actin values.

Experiment	Stock	TRT	ABPV	BQCV	CBPV	DWV-A	DWV-B	IAPV	KBV	LSV
CBPV	*Italian*	*Control*	9 (10)	0 (10)	4 (10)	10 (10)	10 (10)	3 (10)	0 (10)	0 (10)
		PBS	5 (9)	0 (9)	8 (9)	9 (9)	9 (9)	0 (9)	0 (9)	0 (9)
		CBPV	6 (8)	0 (8)	8 (8)	8 (8)	8 (8)	0 (8)	0 (8)	0 (8)
	Pol-Line	*Control*	7 (12)	1 (12)	7 (12)	9 (12)	12 (12)	5 (12)	1 (12)	0 (12)
		PBS	0 (12)	0 (12)	10 (12)	12 (12)	12 (12)	0 (12)	0 (12)	0 (12)
		CBPV	8 (12)	0 (12)	12 (12)	11 (12)	12 (12)	0 (12)	0 (12)	0 (12)
	Russian	*Control*	8 (12)	1 (12)	5 (12)	12 (12)	12 (12)	3 (12)	0 (12)	0 (12)
		PBS	2 (12)	0 (12)	12 (12)	12 (12)	12 (12)	0 (12)	0 (12)	0 (12)
		CBPV	3 (12)	0 (12)	12 (12)	12 (12)	12 (12)	0 (12)	0 (12)	0 (12)
DWV	*Italian*	*Control*	6 (12)	0 (12)	5 (12)	12 (12)	12 (12)	0 (12)	11 (12)	0 (12)
		PBS	2 (11)	0 (11)	6 (11)	11 (11)	11 (11)	0 (11)	0 (11)	0 (11)
		DWV	4 (10)	0 (10)	7 (10)	10 (10)	10 (10)	0 (10)	0 (10)	0 (10)
	Pol-Line	*Control*	7 (12)	1 (12)	7 (12)	12 (12)	12 (12)	1 (12)	11 (12)	1 (12)
		PBS	3 (12)	0 (12)	2 (12)	12 (12)	12 (12)	0 (12)	0 (12)	0 (12)
		DWV	4 (10)	0 (10)	10 (10)	10 (10)	10 (10)	0 (10)	0 (10)	0 (10)
	Russian	*Control*	8 (12)	1 (12)	4 (12)	12 (12)	12 (12)	1 (12)	10 (12)	0 (12)
		PBS	1 (8)	0 (8)	0 (8)	8 (8)	8 (8)	0 (8)	0 (8)	0 (8)
		DWV	4 (11)	0 (11)	9 (11)	11 (11)	11 (11)	0 (11)	0 (11)	0 (11)

Biology Open • Supplementary information
